# Functional impact of cancer patient‐associated Bcl‐xL mutations

**DOI:** 10.1002/mco2.36

**Published:** 2020-10-29

**Authors:** Tiantian Zhang, Joseph HyungJoon Na, Samantha Li, Zhengming Chen, George Zhang, Sharon Pang, Anthony F. Daniyan, Yi Li, Lei Shi, Yi‐Chieh Nancy Du

**Affiliations:** ^1^ Department of Pathology and Laboratory Medicine Weill Cornell Medicine New York New York; ^2^ Division of Biostatistics and Epidemiology, Department of Population Health Sciences Weill Cornell Medicine New York New York; ^3^ Department of Medicine, Sloan Kettering Institute Memorial Sloan Kettering Cancer Center New York New York; ^4^ Lester and Sue Smith Breast Center Baylor College of Medicine Houston Texas; ^5^ Computational Chemistry and Molecular Biophysics Unit, National Institute on Drug Abuse, Intramural Research Program National Institutes of Health Baltimore Maryland

**Keywords:** antiapoptosis, Bcl‐xL, migration, mutations, nucleus

## Abstract

Bcl‐xL, an antiapoptotic protein, is frequently overexpressed in cancer to promote survival of tumor cells. However, we have previously shown that Bcl‐xL promotes migration, invasion, and metastasis independent of its antiapoptotic function in mitochondria. The pro‐metastatic function of Bcl‐xL may require its translocation into the nucleus. Besides overexpression, patient‐associated mutations of Bcl‐xL have been identified in large‐scale cancer genomics projects. Understanding the functions of these mutations will guide the development of precision medicine. Here, we selected four patient‐associated Bcl‐xL mutations, R132W, N136K, R165W, and A201T, to investigate their impacts on antiapoptosis, migration, and nuclear translocation. We found that all four mutation proteins could be detected in both the nucleus and cytosol. Although all four mutations disrupted the antiapoptosis function, one of these mutants, N136K, significantly improved the ability to promote cell migration. These data suggest the importance of developing novel Bcl‐xL inhibitors to ablate both antiapoptotic and pro‐metastatic functions of Bcl‐xL in cancer.

## Graphical abstract

Bcl‐xL is well‐known for its antiapoptotic function in cancer. In this study, we described that four cancer‐associated Bcl‐xL mutations, R132W, N136K, R165W, and A201T, behaved differently in antiapoptosis, migration, and nuclear translocation. N136K and R165W impaired the antiapoptotic function of Bcl‐xL, but still promoted cell migration. In particular, N136K significantly improved the ability to promote cell migration. These data suggested the importance of developing new strategies beyond canonical Bcl‐xL inhibitors to ablate both antiapoptotic and metastatic functions in cancer.

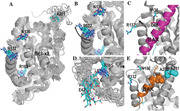

**Abbreviations**
Bcl‐2: B‐cell lymphoma 2
BH: Bcl‐2 homology
EMT: epithelial‐mesenchymal transition
FBS: fetal bovine serum
MOMP: mitochondrial outer membrane permeabilization
N/C: nuclear to cytosol
NMR: nuclear magnetic resonance
PCR: polymerase chain reaction
PNET: pancreatic neuroendocrine tumor
pQ: pQCXIP retroviral vector
TGL: thymidine kinase/GFP/luciferase reporter
SEM: the standard error of the mean
TM: transmembrane
VAF: variant allele frequency
WT: wild‐type

## 1 INTRODUCTION

The intrinsic apoptotic pathway is regulated, in large part, by the mitochondrial outer membrane permeabilization (MOMP); when MOMP occurs, cytochrome c is released and downstream cysteinyl aspartate‐specific proteases (caspases) become activated.^1^ The members of the B‐cell lymphoma 2 (Bcl‐2) family are designated as such due to their Bcl‐2 homology (BH) domains and involvement in apoptosis regulation. The BH domains facilitate the family members’ interactions with each other and can indicate pro‐ or antiapoptotic function. These proteins are divided into three subfamilies: (a) antiapoptotic, (b) pore‐forming or ‘executioner’ (pro‐apoptotic) proteins, and (c) BH3‐only (pro‐apoptotic), based on whether they lead to MOMP (pro‐apoptotic) or inhibit it (antiapoptotic). In healthy cells, antiapoptotic Bcl‐2 proteins hold Bax and Bak, pro‐apoptotic regulator proteins, in check. By the stimulation of apoptotic signals, the BH3‐only proteins promote apoptosis by either activating Bax and Bak or inactivating Bcl‐2, Bcl‐xL, and Mcl‐1.^2^ Subsequently, Bax and Bak are recruited to the mitochondrial outer membrane, where they oligomerize and cause MOMP to release pro‐apoptotic effectors. Antiapoptotic Bcl‐2 family members, including Bcl‐xL, are often overexpressed in a variety of cancers through genetic alterations. Bcl‐xL is well‐known for its antiapoptotic function, which is critical to the development and survival of multicellular organisms.^2^ The function of Bcl‐xL in tumorigenesis has been ascribed to its antiapoptotic activity.

In addition to its antiapoptotic function, other functions of Bcl‐xL have been discovered. Using two engineered Bcl‐xL mutants that cannot bind to Bax and Bak and therefore are defective in antiapoptotic function, we demonstrated that these engineered mutants as well as wild‐type (WT) Bcl‐xL are capable of inducing epithelial‐mesenchymal transition (EMT), migration, invasion, and stemness in both pancreatic neuroendocrine tumor and breast cancer cell lines.^3^, ^4^ WT Bcl‐xL and engineered Bcl‐xL mutants that are defective in antiapoptotic function promote metastasis in spontaneous and xenograft mouse models.^3^, ^4^ These findings suggest that Bcl‐xL's metastatic function is independent of its antiapoptotic function. Other studies reported that overexpression of Bcl‐xL induces EMT in lung cancer cell lines, increases invasiveness of glioma cell lines and, promotes metastasis of breast cancer cell lines in xenograft models.^5^, ^6^, ^7^, ^8^, ^9^ Moreover, overexpression of Bcl‐xL in breast cancer patients is associated with high tumor grade, local invasion into stroma, and nodal metastases.^10^ We have further shown that an engineered Bcl‐xL targeted to the nucleus, but not mitochondria‐bound Bcl‐xL or Bcl‐xL tagged with nuclear export sequence, promotes cell migration, invasion, and EMT in multiple cell lines.^3^

Mutations in proteins play an important role in the onset and development of cancer.^11^, ^12^ Mutations may affect protein folding and stability,^13^, ^14^, ^15^, ^16^, ^17^, ^18^ protein function,^19^, ^20^ and protein‐protein interactions,^21^, ^22^, ^23^, ^24^ as well as protein expression and subcellular localization.^15^, ^16^ Large‐scale cancer genome projects have reported lists of mutations in various tumor types and this information has facilitated subsequent investigations into the functions of these mutant proteins, as well as further therapeutic development of mutation‐specific therapeutics to target the mutant proteins. Cancer patient‐associated mutations of Bcl‐xL have been identified with unknown functional impact. In this study, we aim to investigate whether these cancer patient‐associated mutations of Bcl‐xL affect its functions in antiapoptosis, cell migration, and nuclear translocation. This information will aid therapeutic development to better target Bcl‐xL.

## 2 RESULTS

### 2.1 Selection of four patient‐associated mutations of Bcl‐xL and computational structural analysis

More than 30 mutations of Bcl‐xL have been identified in large‐scale cancer genomic datasets.^25^, ^26^ These mutations could vary greatly in their functional impact, depending on their position in the protein and nature of the mutated amino acid residue. We used a mutation assessor score and variant allele frequency in the tumor samples to select candidate Bcl‐xL mutations for this study. The mutation assessor score represents the functional impact of a missense variant.^12^ Variant allele frequency is “read count supporting mutant base per total read count at that position.”^27^ It is rare that a tumor sample is 100% pure with no normal stromal cells, and is also composed of tumor cells containing the same mutations. However, the higher the variant allele frequency is in the tumor sample, the more likely the mutation is to be a driver mutation rather than a passenger mutation. Therefore, we used a cutoff value of 0.1 for the variant allele frequency in the tumor samples. Four missense mutations, R132W, N136K, R165W, and A201T, matched our selection criteria (Table 1).

**TABLE 1:** Patient‐associated Bcl‐xL mutations with medium mutation assessor score and >0.1 variant allele frequency (VAF) in the tumor samples

Sample ID	Cancer study	AA change	Type	Copy number	Mutation assessor	VAF (T)
DND41_HAEMATOPOIETIC_ AND_LYMPHOID_TISSUE	CCLE	R132W	Missense	Gain	Medium	0.14
						
L428_HAEMATOPOIETIC_AND_ LYMPHOID_TISSUE	CCLE	N136K	Missense	Diploid	Medium	0.44
ESO‐0061	Esophagus (Broad)	R165W	Missense	NA	Medium	0.49
TCGA‐N7‐A4Y0‐01	Uterine CS (TCGA)	A201T	Missense	Gain	Medium	0.26

**FIGURE 1:**
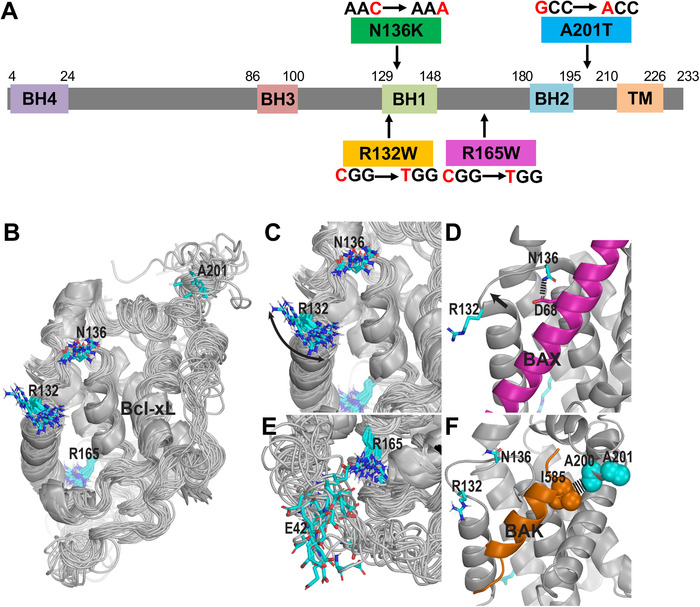
The selected patient‐associated mutations are at critical positions of Bcl‐xL. A, Schematic diagram of Bcl‐xL protein and four patient‐associated mutations, R132W, N136K, R165W, and A201T. BH, Bcl‐2 homology domain; TM, transmembrane domain. B, An overview of the NMR solution structure (including 20 superimposed conformers) of the *apo* state of Bcl‐xL (PDB ID: 6BF2) showing the positions and flexibilities of four residues (in stick representations) that have been found to be mutated in patients. The regions in helical conformations are shown as ribbons. C, A zoom‐in view of R132 and N136 near the ligand binding pocket. The arrow indicates that the side chain of R132 is highly dynamic. D, In binding to BAK (magenta, PDB ID: 3PL7), N136 forms a H‐bond with D68 of Bax, whereas the region near R132 moves outward compared to that in panel B, as indicated by the arrow. E, The side chain of R165 can interact with E42 from the IDR. F, A201 is in the immediate vicinity to A200, which forms a hydrophobic interaction with I585 of BAK. Note that panels C, D, and F are in the same viewing angle as that of panel B

Bcl‐xL contains four distinct BH domains (BH1‐BH4) as well as a transmembrane (TM) region. R132W and N136K are located in the BH1 domain, R165W is located in the loop that is between BH1 and BH2 domains, and A201T is located between the BH2 domain and the C‐terminal TM domain (Figure 1A). We analyzed the positions of these mutations in the context of the hydrophobic ligand binding pocket formed by BH1, BH2, and BH3 domains (Figure 1B). We found that the R132 residue is in close proximity to the hydrophobic pocket, showing significant dynamics in the apo nuclear magnetic resonance (NMR) structures of Bcl‐xL (PDB ID: 6BF2; Figure 1C) and may potentially be involved in the entry of the ligand. The BH1 region in which R132 and N136 are located can make conformational changes upon ligand binding (indicated by the arrow in Figure 1D). In addition, N136 can involve in the ligand binding itself, for example, it forms a direct interaction with D68 of Bax (PDB ID: 3PL7; Figure 1D), however, not with Bak (PDB ID: 1BXL; Figure 1F). R165 faces the intrinsically disordered region (IDR) (PDB ID: 6BF2) and can form a H‐bond with either the backbone or the sidechain of E42 (Figure 1E). The posttranslational modifications of this IDR have recently been found to be able to regulate apoptosis.^28^ A201 is located very next to A200, which is involved in a hydrophobic interaction with the signature “h4” residue of the ligand, such as I585 of BAK^29^ (Figure 1F), whereas in apo structure, this region around A201 is highly flexible (PDB ID: 6BF2).

### 2.2 Overexpression of the Bcl‐xL mutants in MCF7 cells

**FIGURE 2:**
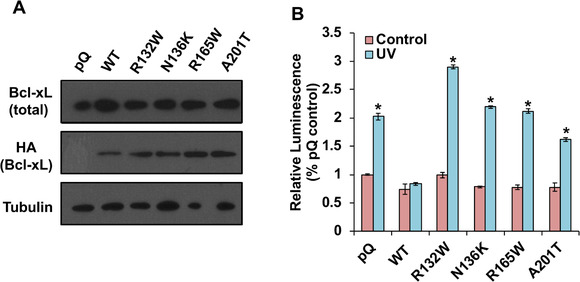
All four cancer‐associated mutations impair the antiapoptotic function of Bcl‐xL. A, Bcl‐xL mutants were overexpressed at similar levels in MCF7/TGL cells. Established cell lines (MCF7/TGL/pQ, MCF7/TGL/HA‐Bcl‐xL, MCF7/TGL/HA‐Bcl‐xL^R132W^, MCF7/TGL/HA‐Bcl‐xL^N136K^, MCF7/TGL/HA‐Bcl‐xL^R165W^, and MCF7/TGL/HA‐Bcl‐xL^A201T^) were maintained with 0.5 µg/mL puromycin and harvested for Western blot analyses of Bcl‐xL and HA‐Bcl‐xL protein levels. α‐Tubulin was used as a loading control. B, Indicated cell lines were treated or untreated with 50 J/m UV. After 3 h, the apoptotic rates were measured by Caspase‐Glo 3/7 Assay System. Results were presented as mean ± the standard error of the mean (SEM). The differences between control condition and UV induced condition were compared by student's *t*‐test in GraphPad Prism. *Statistically significant difference at *P *< .05

To generate these four patient‐associated mutations of Bcl‐xL, we designed primers to make the corresponding missense mutations from WT Bcl‐xL DNA template with N‐terminal HA tag in the pQCXIP retroviral vector (pQ) by polymerase chain reaction (PCR) site‐directed mutagenesis. The four HA‐tagged Bcl‐xL mutants, R132W, N136K, R165W, and A201T, were transfected into a packaging cell line and the viral supernatants were collected to infect MCF7/TGL breast cancer cells, which has low levels of endogenous Bcl‐xL. Stable infected cell lines were selected under puromycin. To examine whether these Bcl‐xL mutants can be expressed properly in these cells, cell lysates were collected for Western blot analysis. WT HA‐tagged Bcl‐xL and the four HA‐tagged Bcl‐xL mutants, R132W, N136K, R165W, and A201T, were expressed well in MCF7/TGL cells (Figure 2A), suggesting that these mutations do not affect protein stability in this cell line.

### 2.3 R132W, N136K, R165W, and A201T reduce the antiapoptotic function of Bcl‐xL

We first investigated whether these four patient‐associated mutations affected the well‐known antiapoptotic function of Bcl‐xL. We treated the cells with ultraviolet (UV) light to induce apoptosis and then measured the activities of caspase 3 and caspase 7 after 3 h. As expected, WT HA‐Bcl‐xL protected cells from UV‐induced apoptosis, and the control pQ vector did not (Figure 2B). We found that all four mutations reduced the antiapoptotic function to different degrees (Figure 2B). Intriguingly, cells overexpressing the R132W mutant protein were even more sensitive to UV radiation than cells overexpressing the control pQ vector.

### 2.4 N136K and R165W still promoted cell migration, whereas R132W and A201T impaired the migration function of Bcl‐xL

**FIGURE 3:**
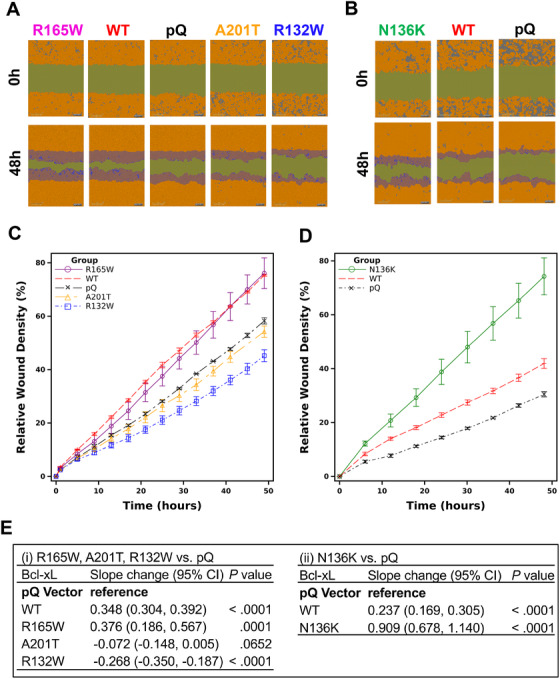
N136K and R165W promoted cell migration, whereas R132W and A201T reduced the migration function of Bcl‐xL. MCF7/TGL cells expressing indicated Bcl‐xL constructs (WT, R132W, N136K, R165W, and A201T) and pQ vector were seeded in 96‐Well ImageLock plates in the concentration of 35 000 cells/100 µL and confluent cells were subjected to automated wound healing assay in IncuCyte. A and B, Images of the wounds were taken every 4 or 6 h for 48 h, and the representative images of each cell line at the starting point (0 h) and the end point (48 h) were shown. C and D, Mean of the relative wound density with SEM of the MCF7/TGL cells expressing indicated Bcl‐xL constructs and pQ vector was plotted over time. The relative wound density was set as 0% at *t* = 0, and 100% when the cell density inside the wound was the same as that outside the initial wound. E, The slopes of the wound healing rates for each Bcl‐xL construct were analyzed by GEE method to test the overall difference of cell migration compared to pQ. CI, confidence interval. All analyses were performed in statistical software SAS Version 9.4 (SAS Institute, Cary, NC)

To investigate whether these four mutants affect the function of Bcl‐xL in promoting cell migration, we performed wound healing assays. MCF7/TGL control cells (pQ) and cells overexpressing WT HA‐Bcl‐xL and the four HA‐Bcl‐xL mutants, R132W, N136K, R165W, and A201T, were grown into a monolayer. After scratch wounds were made, the culture plates were placed into an IncuCyte Zoom machine. The relative wound density was quantified by measuring the spatial cell density in the wound area relative to the spatial cell density outside of the wound area every 4 or 6 h. The relative wound density was set to be 0% at the starting time point, and 100% when the cell density inside the wound was the same as that outside the initial wound (Figure 3). This system was also self‐normalizing for changes in cell density due to cell proliferation. Then, we used GEE method to determine the overall difference in cell migration between cell lines over time. Due to unknown technical difficulties to have all six cell lines grown into a monolayer in the 96‐well ImageLock plates at the same time, we first compared R132W, R165W, and A201T with pQ and WT HA‐Bcl‐xL (Figure 3A, 3C, and 3E), and then compared N136K with pQ and WT HA‐Bcl‐xL (Figure 3B, 3D, and 3E). The change per unit of time in cells expressing the control pQ vector was set as the reference (Figure 3E). We found that cells overexpressing the R132W mutant migrated significantly slower than the pQ group (Figure 3A, 3C, and 3E; *P *< .001) and that cells overexpressing the A201T mutant were not significantly different from the pQ group (Figure 3A, 3C, and 3E; *P *= .0652). Cells overexpressing the R165W mutant migrated significantly faster than the pQ control (Figure 3; *P *< .001) but were comparable to the cells overexpressing the WT counterpart. Importantly, cells overexpressing the N136K mutant were dramatically better in migration than cells overexpressing WT Bcl‐xL protein (Figure 3; *P *< .001). Together, these results suggested that the N136K mutation may have conferred a greater migration potential at the expense of losing the antiapoptotic activity.

### 2.5 All four mutant proteins, as well as WT Bcl‐xL, can be found in both the cytosol and nucleus

**FIGURE 4:**
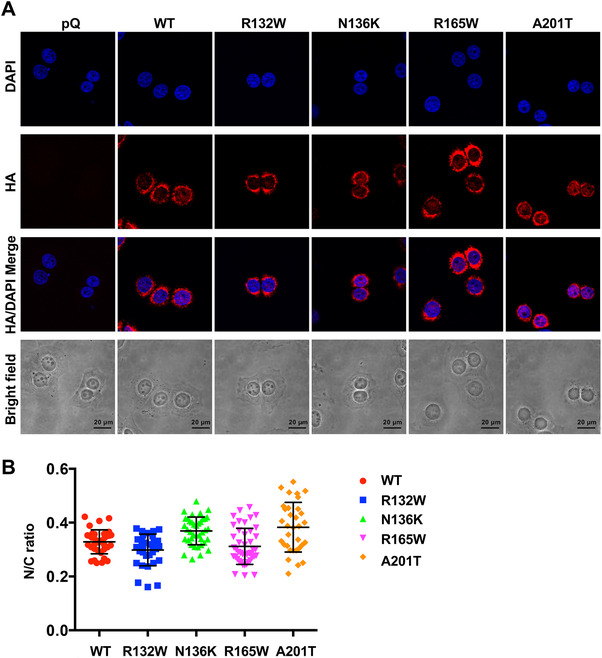
All four Bcl‐xL mutations were still able to translocate into the nucleus. Cell lines (MCF7/TGL/pQ, MCF7/TGL/HA‐Bcl‐xL, MCF7/TGL/HA‐Bcl‐xL^R132W^, MCF7/TGL/HA‐Bcl‐xL^N136K^, MCF7/TGL/HA‐Bcl‐xL^R165W^, and MCF7/TGL/HA‐Bcl‐xL^A201T^) were fixed and stained with anti‐HA antibodies (red) and 4′,6‐diamidino‐2‐phenylindole (DAPI) (blue) and observed using confocal microscopy for subcellular localization of HA‐Bcl‐xL. A, Representative images were shown. Scale bar, 20 µm. Original magnification, ×60. B, Whole cell and the nuclear to cytosol ratio of HA‐Bcl‐xL florescent signals were quantified in at least 40 individual cells per cell line. Results were presented as mean ± SEM. The differences among multiple groups were analyzed by one‐way ANOVA in GraphPad Prism. *Statistically significant difference at *P* < .05

We have demonstrated that the metastatic function of Bcl‐xL is independent of its antiapoptotic function and its residence in the mitochondria.^3^ This novel metastatic function may require its translocation in to the nucleus.^3^ To investigate the subcellular localizations of these four patient‐associated mutations, we performed immunofluorescence staining for the HA tag in Bcl‐xL proteins using anti‐HA antibodies. Cells were counterstained with DAPI (4′,6‐diamidino‐2‐phenylindole) for nucleic acid (nuclear). The nuclear to cytosol (N/C) ratio of WT HA‐Bcl‐xL was .329 (Figure 4 and Table 2). The N/C ratio for R132W and R165W was .298 and .312, respectively (Figure 4 and Table 2), lower than the ratio for WT Bcl‐xL. The N/C ratio for N136K and A201T was .369 and .383, respectively (Figure 4 and Table 2), slightly higher than the ratio for WT Bcl‐xL.

**TABLE 2:** Summary of functions of Bcl‐xL mutants

Bcl‐xL	Antiapoptotic function	Migration function	Subcellular location when overexpressed	Nucleus/cytosol ratio
WT	Yes	Promote migration	Cytosol and nucleus	.329
R132W	No (more sensitive to UV than the pQ vector)	Inhibit migration	Cytosol and nucleus	.298
N136K	No	Promote migration	Cytosol and nucleus	.369
R165W	No	Promote migration	Cytosol and nucleus	.312
A201T	No	Inhibit migration	Cytosol and nucleus	.383

## 3 DISCUSSION

Here, we selected four patient‐associated Bcl‐xL mutations based on the mutation assessor score and variant allele frequency in the tumor samples and characterized their impacts on antiapoptosis, migration, and nuclear translocation. Because the variant allele frequencies of these four patient‐associated Bcl‐xL mutations range from 0.14 to 0.49, WT Bcl‐xL protein could still be present in the same tumor cells. Therefore, we characterized these mutations by expressing them in MCF7 cells, which has low endogenous Bcl‐xL levels and can express the different mutant proteins to similar levels. We also ectopic expressed these mutant proteins in other two cell lines, but they could not be expressed to similar levels. It is possible that some of these exogenous proteins were unstable in certain cell lines.

It is known that BH1, BH2, and BH3 domains of Bcl‐xL create a hydrophobic groove capable of binding BH3‐only pro‐apoptotic proteins, inhibiting their activity and leading to a pro‐survival phenotype.^29^ We found that all four mutations, R132W, N136K, R165W, and A201T, impair Bcl‐xL's antiapoptotic function. R132W and N136K are located in the BH1 domain, which is known to be important for the antiapoptosis function. The reason that R132W was more sensitive to UV‐induced apoptosis is likely due to the potential involvement of the R132 residue in the entry of Bcl‐xL ligands such as Bax and Bak. N136 forms a direct interaction with Bax, not with Bak, suggesting that N136K is not able to interact with Bax to execute its antiapoptotic function. Because R165W and A201T are not located in the BH domains, the reduction in the antiapoptotic function was unexpected. Combining our functional analysis and computational structural analysis, the results indicate that these two residues, R165 and A201, although located in different loops regions, are also important for Bcl‐xL's antiapoptotic function.

The C‐terminal TM domain of Bcl‐xL specifically targets Bcl‐xL to the mitochondrial outer membrane,^30^ so Bcl‐xL translocates to the nucleus by an active mechanism through specific carriers in cancer cells. Because none of these four mutations completely abolish the nuclear translocation of Bcl‐xL, these residues are not critical for nuclear input of Bcl‐xL. However, R132W had the least nuclear Bcl‐xL expression among the four mutants. Further experiments are required to investigate whether the R132W mutation of Bcl‐xL weakens its interaction with the carrier proteins that translocate Bcl‐xL into the nucleus. Although some R132W and A201T mutant proteins were still located in the nucleus, they both significantly impaired the migration function. These data suggest that R132W and A201T mutant proteins cannot interact with the downstream effectors to promote migration. The possible change of its overall protein structure in A201T may explain why this mutation disrupts both functions in antiapoptosis and cell migration. Whether this A201T mutation acquires any new function in cancer requires further study.

N136K and R165W have the highest variant allele frequency among these four mutations, suggesting that they are more likely to be driver mutations in tumorigenesis. Intriguingly, tumor cells harboring N136K or R165W lost antiapoptotic function, but they still possessed migration‐promoting function. Importantly, the N136K mutation boosted the migration potential, highlighting its possible significance in cancer invasion and distant spread. The impact of these mutations on metastasis in vivo requires further investigation. These N136K and R165W mutations could be a great tool to develop novel therapeutics against the metastatic function of Bcl‐xL. In summary, the characterization of patient‐associated Bcl‐xL mutations reveals the importance of developing new strategies beyond canonical Bcl‐xL inhibitors to ablate both antiapoptotic and metastatic functions of Bcl‐xL in cancer.

## 4 MATERIAL AND METHODS

### 4.1 Generation of Bcl‐xL mutations and cell lines

MCF7/TGL cell line was generated by the infection with virus carrying thymidine kinase/*GFP*/luciferase reporter (TGL).^31^ GFP‐positive cells were sorted using BD Biosciences FACS‐DiVa Cell Sorter to generate pure MCF7/TGL cells. Cells were cultured in DMEM supplemented with 10% fetal bovine serum, 0.2 mM L‐glutamine, and 1% penicillin/streptomycin.

Bcl‐xL mutation DNAs (R132W, N136K, R165W, and A201T) were generated using the QuickChange Lightning Site‐Directed Mutagenesis kit (Agilent Technology) following the manufacturer's instruction and verified by sequencing. Plasmid DNAs (pQCXIP [pQ]), pQCXIP‐HA‐Bcl‐xL, pQCXIP‐HA‐Bcl‐xL^R132W^, pQCXIP‐HA‐Bcl‐xL^N136K^, pQCXIP‐HA‐Bcl‐xL^R165W^, and pQCXIP‐HA‐Bcl‐xL^A201T^) were transfected into H29 cells^32^ using Lipofectamine 3000 Reagent and P3000 Reagent (Invitrogen) to generate retroviral supernatant. MCF7/TGL was infected with each retroviral supernatant at 50% confluence in 6‐cm dish, selected with puromycin 72 h postinfection, and maintained with 0.5 µg/mL puromycin.

### 4.2 Western blot

Cells (MCF7/TGL/pQ, MCF7/TGL/HA‐Bcl‐xL, MCF7/TGL/HA‐Bcl‐xL^R132W^, MCF7/TGL/HA‐Bcl‐xL^N136K (Clone 5)^, MCF7/TGL/HA‐Bcl‐xL^R165W^, and MCF7/TGL/HA‐Bcl‐xL^A201T^) were lysed in RIPA buffer (0.1% SDS, 1% Triton X‐100, 0.5% sodium deoxycholate, 25 mM Tris [pH 8.0], 150 mM NaCl, and 1 mM EDTA) supplemented with protease and phosphatase inhibitors (Roche). Protein samples were quantified by Bradford assay (Bio‐Rad), separated through SDS‐PAGE, transferred to nitrocellulose membranes, and stained with Ponceau S. Blots were then incubated in 5% nonfat milk in TBST for 2 h, probed with primary antibodies Bcl‐xL (1:1000, Cell Signaling Technology, 2764), HA (1:1000, Cell Signaling Technology, 2367), or α‐tubulin (1:1000, Sigma, T5168) overnight at 4°C, washed three times with TBST for a total of 30 min, and probed with horseradish peroxidase‐conjugated secondary antibodies for 1 h. Signals were detected by enhanced chemical luminescence (Pierce).

### 4.3 Apoptosis assay

A total of 1 × 10^4^ cells (MCF7/TGL/pQ, MCF7/TGL/HA‐Bcl‐xL, MCF7/TGL/HA‐Bcl‐xL^R132W^, MCF7/TGL/HA‐Bcl‐xL^N136K (Clone 5)^, MCF7/TGL/HA‐Bcl‐xL^R165W^, and MCF7/TGL/HA‐Bcl‐xL^A201T^) were seeded in 100‐µL culture medium in 96‐well plate. After overnight incubation, cells either remained untreated or were treated with 50 J/m UV. Three hours after UV treatment, apoptosis was measured by Caspase‐Glo 3/7 Assay System (Promega, G8090) following the manufacturer's instruction. The luminance was read by the EnVision Multilabel Plate Reader (PerkinElmer).

### 4.4 Immunofluorescent analysis

Cells (MCF7/TGL/pQ, MCF7/TGL/HA‐Bcl‐xL, MCF7/TGL/HA‐Bcl‐xL^R132W^, MCF7/TGL/HA‐Bcl‐xL^N136K (Clone 5)^, MCF7/TGL/HA‐Bcl‐xL^R165W^, and MCF7/TGL/HA‐Bcl‐xL^A201T^) were cultured on glass coverslips for 24 h before fixation in 4% paraformaldehyde in PBS for 10 min. After permeabilizing and blocking in 0.5% BSA in PBS with 0.025% Triton X‐100, 0.02% NaN_3_, and 0.3 mM DAPI for 2 h, coverslips were incubated with primary antibody (mouse anti‐HA 1:200; Cell Signaling Technology, 2367) overnight at 4°C. The coverslips were then washed four times with PBS for 5 min each, followed by incubation with Alexa Fluor 647 goat antimouse antibody (1:400; Life Technologies, A21236) at room temperature in the dark for 2 h, and were washed four times with PBS for 5 min each. Coverslips were mounted with VectaShield mounting medium (Vector Labs) and cells were examined using confocal microscopy (Olympus FLUOVIEW FV10i). The total, nuclear, and cytoplasmic immunofluorescence signals of more than 40 cells per cell line from a minimum of 10 separate images were quantified using Fiji ImageJ (version 1.51p).

### 4.5 Wound healing migration assay

For each cell line (MCF7/TGL/pQ, MCF7/TGL/HA‐Bcl‐xL, MCF7/TGL/HA‐Bcl‐xL^R132W^, MCF7/TGL/HA‐Bcl‐xL^N136K (Clone 5)^, MCF7/TGL/HA‐Bcl‐xL^R165W^, and MCF7/TGL/HA‐Bcl‐xL^A201T^), 3.5 × 10^5^ cells in 100 µL media were seeded into each well of a 96‐well ImageLock tissue culture plate (n = 3) (Essen BioScience, 4379) and incubated overnight in standard cell incubator at 37°C. The plate was then removed from the incubator, and WoundMaker was used to create scratch wound in all wells of the 96‐well ImageLock plate. Then, media from each well was aspirated and gently washed with culture media to prevent dislodged cells from settling and reattaching. After washing, 100 µL of culture media was added and placed into the IncuCyte Zoom machine. The IncuCyte Zoom Plate Map Editor 2016A software was used to set the scan type to Scratch Wound and Wide Mode and set the image acquisition interval to repeat scan every 4 or 6 h for 48 h automatically. The processed data for relative wound density for the cell lines after each scan were used to compare the rate of migration. The IncuCyte software quantified “relative wound density” by measuring the spatial cell density in the wound area relative to the spatial cell density outside of the wound area at every time point. “Relative wound density” was set to be 0% at *t* = 0, and 100% when the cell density inside the wound became the same as the cell density outside the initial wound.

### 4.6 Statistical analysis

Differences between two groups were compared by Student's *t*‐test or Wilcoxon rank‐sum test, as appropriate. Differences among multiple groups were compared using one‐way ANOVA. GEE method was used to test the overall difference of cell migration between two cell lines over time. All analyses were performed in GraphPad Prism or SAS9.4 (SAS Institute, Cary, NC).

## CONFLICT OF INTEREST

The authors declare no conflict of interest.

## AUTHOR CONTRIBUTIONS

TZ, JHN, SL, GZ, SP, and AFD designed and performed the experiments and analyzed the data. ZC performed statistical analysis. LS performed computational structural analysis. TZ and Y‐CND wrote the manuscript. All authors discussed the results and commented on the manuscript.

## Data Availability

The authors confirm that the data supporting the findings of this study are available within the article.

## References

[1] Riedl SJ , Salvesen GS . The apoptosome: signalling platform of cell death. Nat Rev Mol Cell Biol. 2007;8(5):405‐413. 17377525 10.1038/nrm2153

[2] Youle RJ , Strasser A . The Bcl‐2 protein family: opposing activities that mediate cell death. Nat Rev Mol Cell Biol. 2008;9(1):47‐59. 18097445 10.1038/nrm2308

[3] Choi S , Chen Z , Tang LH , et al. Bcl‐xL promotes metastasis independent of its anti‐apoptotic activity. Nat Commun. 2016;7:10384. 26785948 10.1038/ncomms10384 PMC4735924

[4] Du YC , Lewis BC , Hanahan D , Varmus H . Assessing tumor progression factors by somatic gene transfer into a mouse model: Bcl‐xL promotes islet tumor cell invasion. PLoS Biol. 2007;5(10):e276. 17941720 10.1371/journal.pbio.0050276 PMC2020504

[5] Ho JN , Kang GY , Lee SS , et al. Bcl‐xL and STAT3 mediate malignant actions of gamma‐irradiation in lung cancer cells. Cancer Sci. 2010;101(6):1417‐1423. 20331635 10.1111/j.1349-7006.2010.01552.x PMC11159096

[6] Weiler M , Bahr O , Hohlweg U , et al. Bcl‐xL: time‐dependent dissociation between modulation of apoptosis and invasiveness in human malignant glioma cells. Cell Death Differ. 2006;13(7):1156‐1169. 16254573 10.1038/sj.cdd.4401786

[7] Espana L , Fernandez Y , Rubio N , Torregrosa A , Blanco J , Sierra A . Overexpression of Bcl‐xL in human breast cancer cells enhances organ‐selective lymph node metastasis. Breast Cancer Res Treat. 2004;87(1):33‐44. 15377849 10.1023/B:BREA.0000041579.51902.89

[8] Fernandez Y , Espana L , Manas S , Fabra A , Sierra A . Bcl‐xL promotes metastasis of breast cancer cells by induction of cytokines resistance. Cell Death Differ. 2000;7(4):350‐359. 10773819 10.1038/sj.cdd.4400662

[9] Rubio N , Espana L , Fernandez Y , Blanco J , Sierra A . Metastatic behavior of human breast carcinomas overexpressing the Bcl‐xL gene: a role in dormancy and organospecificity. Lab Invest. 2001;81(5):725‐734. 11351044 10.1038/labinvest.3780281

[10] Keitel U , Scheel A , Thomale J , et al. Bcl‐xL mediates therapeutic resistance of a mesenchymal breast cancer cell subpopulation. Oncotarget. 2014;5(23):11778‐11791. 25473892 10.18632/oncotarget.2634 PMC4322974

[11] Hanahan D , Weinberg RA . Hallmarks of cancer: the next generation. Cell. 2011;144(5):646‐674. 21376230 10.1016/j.cell.2011.02.013

[12] Reva B , Antipin Y , Sander C . Predicting the functional impact of protein mutations: application to cancer genomics. Nucleic Acids Res. 2011;39(17):e118. 21727090 10.1093/nar/gkr407 PMC3177186

[13] Ode H , Matsuyama S , Hata M , et al. Computational characterization of structural role of the non‐active site mutation M36I of human immunodeficiency virus type 1 protease. J Mol Biol. 2007;370(3):598‐607. 17524421 10.1016/j.jmb.2007.04.081

[14] Lorch M , Mason JM , Sessions RB , Clarke AR . Effects of mutations on the thermodynamics of a protein folding reaction: implications for the mechanism of formation of the intermediate and transition states. Biochemistry. 2000;39(12):3480‐3485. 10727243 10.1021/bi9923510

[15] Lorch M , Mason JM , Clarke AR , Parker MJ . Effects of core mutations on the folding of a beta‐sheet protein: implications for backbone organization in the I‐state. Biochemistry. 1999;38(4):1377‐1385. 9931001 10.1021/bi9817820

[16] Alfalah M , Keiser M , Leeb T , Zimmer KP , Naim HY . Compound heterozygous mutations affect protein folding and function in patients with congenital sucrase‐isomaltase deficiency. Gastroenterology. 2009;136(3):883‐892. 19121318 10.1053/j.gastro.2008.11.038

[17] Koukouritaki SB , Poch MT , Henderson MC , et al. Identification and functional analysis of common human flavin‐containing monooxygenase 3 genetic variants. J Pharmacol Exp Ther. 2007;320(1):266‐273. 17050781 10.1124/jpet.106.112268

[18] De Cristofaro R , Carotti A , Akhavan S , et al. The natural mutation by deletion of Lys9 in the thrombin A‐chain affects the pKa value of catalytic residues, the overall enzyme's stability and conformational transitions linked to Na+ binding. FEBS J. 2006;273(1):159‐169. 16367756 10.1111/j.1742-4658.2005.05052.x

[19] Yamada Y , Banno Y , Yoshida H , et al. Catalytic inactivation of human phospholipase D2 by a naturally occurring Gly901Asp mutation. Arch Med Res. 2006;37(6):696‐699. 16824927 10.1016/j.arcmed.2006.01.006

[20] Takamiya O , Seta M , Tanaka K , Ishida F . Human factor VII deficiency caused by S339C mutation located adjacent to the specificity pocket of the catalytic domain. Clin Lab Haematol. 2002;24(4):233‐238. 12181027 10.1046/j.1365-2257.2002.00449.x

[21] Jones R , Ruas M , Gregory F , et al. A CDKN2A mutation in familial melanoma that abrogates binding of p16INK4a to CDK4 but not CDK6. Cancer Res. 2007;67(19):9134‐9141. 17909018 10.1158/0008-5472.CAN-07-1528

[22] Ung MU , Lu B , McCammon JA . E230Q mutation of the catalytic subunit of cAMP‐dependent protein kinase affects local structure and the binding of peptide inhibitor. Biopolymers. 2006;81(6):428‐439. 16365849 10.1002/bip.20434

[23] Rignall TR , Baker JO , McCarter SL , et al. Effect of single active‐site cleft mutation on product specificity in a thermostable bacterial cellulase. Appl Biochem Biotechnol. 2002;98‐100:383‐394. 10.1385/abab:98-100:1-9:383 12018266

[24] Hardt M , Laine RA . Mutation of active site residues in the chitin‐binding domain ChBDChiA1 from chitinase A1 of *Bacillus circulans* alters substrate specificity: use of a green fluorescent protein binding assay. Arch Biochem Biophys. 2004;426(2):286‐297. 15158679 10.1016/j.abb.2004.03.017

[25] Cerami E , Gao J , Dogrusoz U , et al. The cBio cancer genomics portal: an open platform for exploring multidimensional cancer genomics data. Cancer Discov. 2012;2(5):401‐404. 22588877 10.1158/2159-8290.CD-12-0095 PMC3956037

[26] Gao J , Aksoy BA , Dogrusoz U , et al. Integrative analysis of complex cancer genomics and clinical profiles using the cBioPortal. Sci Signal. 2013;6(269):pl1. 23550210 10.1126/scisignal.2004088 PMC4160307

[27] Strom SP . Current practices and guidelines for clinical next‐generation sequencing oncology testing. Cancer Biol Med. 2016;13(1):3‐11. 27144058 10.28092/j.issn.2095-3941.2016.0004 PMC4850126

[28] Follis AV , Llambi F , Kalkavan H , et al. Regulation of apoptosis by an intrinsically disordered region of Bcl‐xL. Nat Chem Biol. 2018;14(5):458‐465. 29507390 10.1038/s41589-018-0011-x PMC5899648

[29] Lee EF , Fairlie WD . The structural biology of Bcl‐xL. Int J Mol Sci. 2019;20(9):2234. 10.3390/ijms20092234 PMC6540150 31067648

[30] Kaufmann T , Schlipf S , Sanz J , Neubert K , Stein R , Borner C . Characterization of the signal that directs Bcl‐xL, but not Bcl‐2, to the mitochondrial outer membrane. J Cell Biol. 2003;160(1):53‐64. 12515824 10.1083/jcb.200210084 PMC2172731

[31] Ponomarev V , Doubrovin M , Serganova I , et al. A novel triple‐modality reporter gene for whole‐body fluorescent, bioluminescent, and nuclear noninvasive imaging. Eur J Nucl Med Mol Imaging. 2004;31(5):740‐751. 15014901 10.1007/s00259-003-1441-5

[32] Zhao Z , Condomines M , van der Stegen SJC , et al. Structural design of engineered costimulation determines tumor rejection kinetics and persistence of CAR T cells. Cancer Cell. 2015;28(4):415‐428. 26461090 10.1016/j.ccell.2015.09.004 PMC5003056

